# Effect of corticosteroids and hyaluronic acid injections on knee osteoarthritis trajectory

**DOI:** 10.1002/jeo2.70333

**Published:** 2025-08-05

**Authors:** Alessandro Bensa, Luca Bianco Prevot, Giuseppe M. Peretti, Giuseppe Filardo

**Affiliations:** ^1^ Lugano Switzerland; ^2^ Faculty of Biomedical Sciences Università della Svizzera Italiana Lugano Switzerland; ^3^ Residency Program in Orthopedics and Traumatology University of Milan Milan Italy; ^4^ IRCCS Ospedale Galeazzi ‐ S. Ambrogio Milan Italy; ^5^ Department of Biomedical Sciences for Health University of Milan Milan Italy

**Keywords:** corticosteroids, hyaluronic acid, intra‐articular, knee, osteoarthritis

## Abstract

**Purpose:**

Knee osteoarthritis (OA) is one of the most prevalent orthopaedic pathologies. Intra‐articular injections represent a common option to manage this condition. The aim of this study was to quantify and compare the effectiveness of corticosteroids (CS) and hyaluronic acid (HA) in affecting the knee OA trajectory over time.

**Methods:**

Patients were selected from the Osteoarthritis Initiative database, a prospective, multicentre, longitudinal, observational study, including 254 knees who received CS or HA injections. For each patient, demographic characteristics, Kellgren–Lawrence (KL) grade, joint space narrowing (JSN) of the medial and lateral compartments, knee swelling, visual analogue scale (VAS) for pain, Western Ontario and McMaster Universities Osteoarthritis Index (WOMAC), and subsequent prosthesis implantation were analysed. Patients were followed from baseline to 36 months of follow‐up. Clinical improvements were evaluated according to the minimal clinically important difference (MCID): VAS = 1.4, WOMAC = 6.4.

**Results:**

Both CS and HA groups showed no overall clinical worsening from the baseline to the 36‐month evaluation. On the other hand, they both presented a worsening at short‐ and long‐term of KL OA grade (CS: *p* < 0.001 and *p* = 0.018; HA: *p* = 0.042 and *p* = 0.012) and medial JSN (CS: *p* < 0.001 and *p* = 0.009, HA: *p* = 0.033 and *p* = 0.003), while lateral JSN deteriorated only in the CS group at short‐term (*p* = 0.030). The analysis of patients obtaining an improvement exceeding the MCID showed that CS outperformed HA at short‐term for WOMAC (44.9% vs. 29.0%, *p* = 0.022) with a tendency also for VAS (44.3% vs. 31.9%, *p* = 0.086). However, only HA provided a clinically relevant long‐term improvement of WOMAC (*p* = 0.014, MD = 6.7) and VAS (*p* = 0.024, MD = 1.2). The analysis of patients requiring total knee arthroplasty (TKA) before the end of the study did not show differences between CS and HA.

**Conclusions:**

The symptomatic trajectory after 36 months showed no worsening in knee OA patients undergoing intra‐articular injections and different benefits based on the treatments: CS offered clinically relevant benefits compared to HA at short‐term while HA provided superior functional improvement at long‐term, with no differences between the two treatments of radiographic OA evolution, knee swelling, and progression to TKA.

**Level of Evidence:**

Level II.

**Abbreviations**
AAOS: American Academy of Orthopaedic Surgeons
ACR: American College of Rheumatology
BMI: body mass index
CS: corticosteroids
ESCEO: European Society for Clinical and Economic Aspects of Osteoporosis and Osteoarthritis
HA: hyaluronic acid
JSN: joint space narrowing
KL: Kellgren–Lawrence
MCID: minimal clinically important difference
NSAIDs: nonsteroidal anti‐inflammatory drugs
OA: osteoarthritis
OAI: Osteoarthritis Initiative
OARSI: Osteoarthritis Research Society International
TKA: total knee arthroplasty
VAS: visual analogue scale
WOMAC: Western Ontario and McMaster Universities Osteoarthritis Index

## INTRODUCTION

Knee osteoarthritis (OA) has an estimated global prevalence of 23% in individuals aged over 40 years and is further increasing, with a heavy burden on healthcare systems worldwide [17, 18, 31, 38]. The manifestations of knee OA include symptoms such as pain, swelling, and functional limitation, ultimately leading to disability in both work‐related tasks and daily life activities [1]. Nonsurgical management of knee OA includes weight loss, physical therapies, dietary supplements, and nonsteroidal anti‐inflammatory drugs (NSAIDs) [22, 33]. When these conservative therapies are no longer effective, intra‐articular injective treatments can represent a minimally invasive option to offer pain relief and functional improvement, with the aim of avoiding or delaying the need for more invasive surgical solutions.

Injective treatments aim at providing clinical improvement by restoring joint homoeostasis thanks to the direct local administration of therapeutic agents [23, 39]. This maximises the intra‐articular product concentration, thereby optimising its therapeutic potential while concurrently minimising systemic exposure and associated side effects [39]. Among the available options for intra‐articular injection, corticosteroids (CS) and hyaluronic acid (HA) represent the most used ones in the clinical practice, being the only injective products currently recommended by major international societies' guidelines [6, 15, 16, 28]. CS have been used for decades in the treatment of symptomatic knee OA, exploiting their anti‐inflammatory effects to provide symptomatic relief [8, 37]. HA represents another widely used option for knee OA thanks to its joint lubrication and shock absorbency properties leading to pain relief and functional improvement [40]. Despite the longstanding and widespread use of these two products, there is still a lack of consensus on the superiority of one approach over the other, fuelling an ongoing debate on the product offering the greatest benefits for knee OA patients.

The aim of this study was to quantify and compare the effectiveness of CS and HA in affecting the knee OA trajectory over time.

## MATERIALS AND METHODS

### Study design

The subjects analysed were selected from the Osteoarthritis Initiative (OAI), a prospective, multicentre, longitudinal, open‐access observational study on 4796 patients. The aim of the OAI was to investigate the knee OA trajectory as well as the risk factors for its onset and progression. Data used in the preparation of the current article are available for public access at http://www.oai.ucsf.edu/. The OAI database provides access to a considerable amount of information of the enroled patients, including radiographic data, clinical data, pain and function questionnaires including the Western Ontario and McMaster Universities Osteoarthritis Index (WOMAC) and visual analogue scale (VAS) for pain [2, 34]. An algorithm was designed using the Python 3.9 Pandas library which made it possible to identify OA patients who performed an intra‐articular injection of CS or HA. Patients were included in the analysis if they answered ‘yes’ to the question: ‘Have you had a cortisone injection in the past 6 months?’ or ‘Have you had a HA injection in the past 6 months?’. Patients in the control cohort of the OAI database (no symptoms and no radiographic tibial‐femoral or patello‐femoral OA in either knee and no OA risk factors), those not receiving CS or HA injections, as well as patients who received previous intra‐articular injection in the studied knee were excluded. The baseline was defined as the follow‐up preceding the report of the injection within a timeframe of 6 months to 1 year before the intra‐articular injection. The time point when patients reported receiving an intra‐articular injection in the past 6 months was considered the early postinjection follow‐up. A second time point, at 2 years postinjection, was designated as the long‐term follow‐up. For each patient, the following baseline data were collected: age, sex, body mass index (BMI), Kellgren–Lawrence (KL) OA grade and joint space narrowing (JSN) of the medial and lateral compartments on postero‐anterior fixed flexion knee radiographs, WOMAC score, VAS pain, the presence of knee swelling, and patients requiring total knee arthroplasty (TKA) before the end of the study. Clinical score improvements were compared to the minimal clinically important difference (MCID), defined as the smallest difference perceived as important by the average patient, based on literature values: VAS = 1.4 and WOMAC = 6.4 [3, 36].

### Statistical analysis

Continuous data were expressed as mean and standard deviation of the mean, while categorical data were expressed as frequency and percentages. The Shapiro–Wilk test was performed to test the normality of continuous variables. The Levene test was used to assess the homoscedasticity of the data. The difference between groups was assessed using one‐way analysis of variance (ANOVA) when the scores were normally distributed and homoscedastic, otherwise the Mann–Whitney nonparametric test was used.

Repeated measures GLM with post‐hoc Sidak correction for multiple comparisons was performed to compare the clinical outcomes at different follow‐ups. For all tests *p* < 0.05 was considered significant. The statistical analysis was performed using SPSS v.19.0 (IBM Corp.).

## RESULTS

The OAI database search according to the described process and criteria allowed to identify 254 knees for the analysis. The characteristics of the patients included are detailed in Table 1. The two groups were comparable in terms of baseline characteristics. The only characteristic presenting a statistically significant difference at baseline was the sex distribution, with HA presenting a greater men percentage than CS (53.6% vs. 34.1%, *p* = 0.006).

**Table 1:** Data are displayed as mean ± standard deviation.

	CS	HA	TOT
Knees	185	69	254
Women (%)	122 (65.9%)	32 (46.4%)	154 (60.6%)
Men (%)	63 (34.1%)	37 (53.6%)	100 (39.4%)
Age (years)	66.4 ± 9.2	64.8 ± 8.3	66.0 ± 9.0
BMI (kg/m²)	29.5 ± 4.5	30.2 ± 4.6	29.7 ± 4.5
KL	1.5 ± 1.1	1.5 ± 1.3	1.5 ± 1.2

Abbreviations: BMI, body mass index; CS, corticosteroids; HA, hyaluronic acid; KL, Kellgren–Lawrence.

### Intragroup analysis

**Figure 1:**
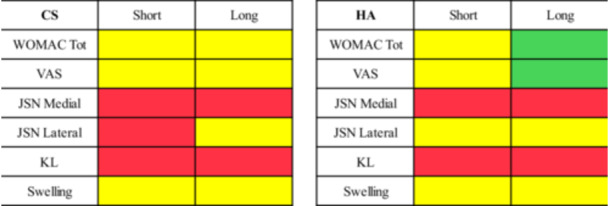
Intragroup analysis of outcomes improvement for corticosteroids (CS) and hyaluronic acid (HA) from baseline to short‐term and long‐term follow‐ups. Green: statistically significant improvement; yellow: no statistical difference; red: statistically significant worsening. JSN, joint space narrowing; KL, Kellgren–Lawrence; VAS, visual analogue scale for pain; WOMAC, Western Ontario and McMaster Universities Osteoarthritis Index.

The overall results are reported in Figure 1. More in detail:

#### WOMAC

The intragroup analysis of the WOMAC score showed that CS did not provide a significant improvement from baseline to any of the two follow‐ups, while HA provided a statistically significant improvement at long‐term follow‐up (*p* = 0.014, MD = 6.7). This improvement exceeded the 6.4 MCID.

#### VAS

The intragroup analysis of VAS scale showed that CS did not provide a significant improvement from baseline to any of the two follow‐ups, while HA provided a statistically significant improvement at long‐term follow‐up (*p* = 0.024, MD = 1.2). This improvement did not exceed the 1.4 MCID.

#### KL

The intragroup analysis of KL showed a statistically significant worsening at both short‐ and long‐term follow‐ups both in the CS group (*p* < 0.001 and *p* = 0.018, respectively) and in the HA group (*p* = 0.042 and *p* = 0.012, respectively).

#### Medial JSN

The intragroup analysis of medial JSN showed a statistically significant worsening at both short‐ and long‐term follow‐ups both in the CS group (*p* < 0.001 and *p* = 0.009, respectively) and in the HA group (*p* = 0.033 and *p* = 0.003, respectively).

#### Lateral JSN

The intragroup analysis of lateral JSN showed a statistically significant worsening at short‐term follow‐up in the CS group (*p* = 0.030), with no difference at long‐term follow‐up, as well as in any follow‐up in the HA group.

#### Swelling

The intragroup analysis of knee swelling did not show any statistically significant change at any follow‐up in both the CS and HA groups.

### Intergroup analysis

#### WOMAC

**Figure 2:**
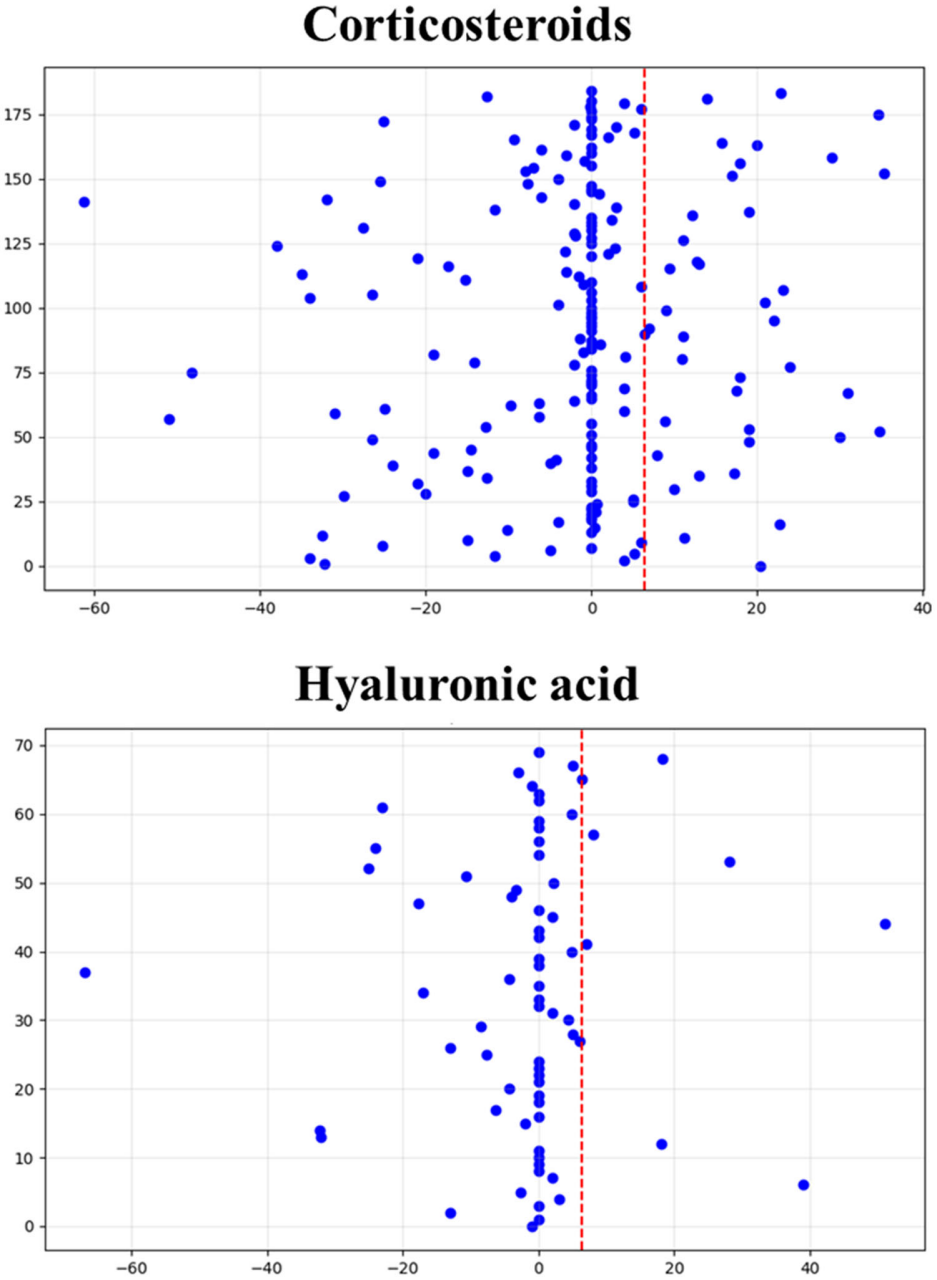
Graphic representation of the amount of patients reaching a Western Ontario and McMaster Universities Osteoarthritis Index (WOMAC) improvement superior to the minimal clinically important difference cut‐off of 6.4 points for the corticosteroids (CS) and hyaluronic acid (HA) groups. CS obtained statistically significant better results than HA at short‐term follow‐up (44.9% vs. 29.0%, *p* = 0.022).

The intergroup analysis of the WOMAC score showed a tendency towards a greater improvement in the HA group at long‐term follow‐up compared to CS (*p* = 0.082, MD = 0.8). This improvement did not exceed the 6.4 MCID. The analysis of the number of patients obtaining an improvement superior to the MCID (Figure 2) showed that CS (44.9%) outperformed HA (29.0%) at short‐term follow‐up (*p* = 0.022), while no difference was shown at long‐term follow‐up.

#### VAS

**Figure 3:**
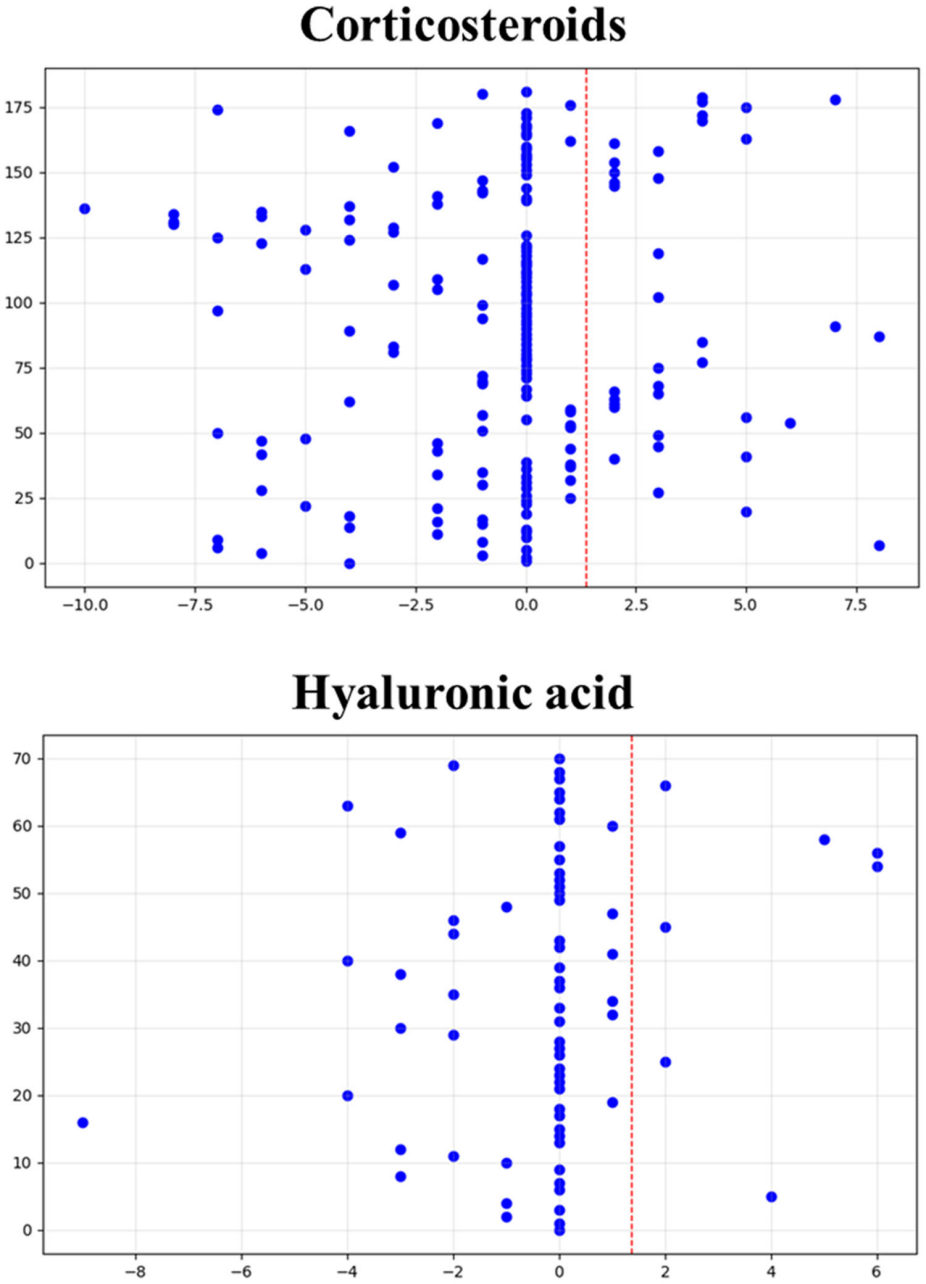
Graphic representation of the amount of patients reaching a visual analogue scale (VAS) improvement superior to the minimal clinically important difference cut‐off of 1.4 points for the corticosteroids (CS) and hyaluronic acid (HA) groups. CS showed a tendency to have better results than HA at short‐term follow‐up (44.3% vs. 31.9%, *p* = 0.086).

The intergroup analysis of VAS improvement did not show any statistically significant difference between CS and HA at any follow‐up. The analysis of the number of patients obtaining an improvement superior to the MCID (Figure 3) showed that CS (44.3%) had a tendency to outperform HA (31.9%) at short‐term follow‐up (*p* = 0.086), while no difference was shown at long‐term follow‐up.

#### KL

The intergroup analysis of KL did not show any statistically significant difference between CS and HA at any follow‐up.

#### Medial JSN

The intergroup analysis of medial JSN did not show any statistically significant difference between CS and HA at any follow‐up.

#### Lateral JSN

The intergroup analysis of lateral JSN did not show any statistically significant difference between CS and HA at any follow‐up.

#### Swelling

The intergroup analysis of knee swelling did not show any statistically significant difference between CS and HA at any follow‐up.

#### TKA

The intergroup analysis of the number of patients requiring TKA before the end of the study did not show any statistically significant difference between CS (3/185, 1.6%) and HA (2/69, 2.9%).

## DISCUSSION

The main finding of this study is that the symptomatic trajectory after 36 months showed no worsening in knee OA patients undergoing intra‐articular injections and different benefits based on the treatments: CS offered clinically relevant benefits compared to HA at short‐term while HA provided superior functional improvement at long‐term, with no differences in terms of radiographic OA evolution, knee swelling, and progression to TKA.

The OAI database documented the trajectory of a large group of knee OA patients presenting a wide range of symptoms and degeneration level. Among these, a subgroup suffered from symptoms requiring the use of intra‐articular injections within the study timeline. Of note, this study analysed a large time‐frame which significantly exceed what commonly reported as the therapeutic window of both products. Accordingly, the long‐term data may be influenced by and interpreted as an effect of the well‐known regression to the mean. The regression to the mean refers to the tendency for extreme measurements to move closer to the average when measured again because these extreme measurements are more likely to be affected by variance. Accordingly, patients are eligible for and seek treatment at a moment in time when they score high on the outcome measures, while follow‐up evaluations may thus represent moments with better symptoms during the physiologic variations of a chronic disease [21]. These retests will therefore regress toward the mean, independent of treatment effects. Patients seeking treatment in their peak symptomatic phases receive therapeutic indications with an increasing level of invasiveness up to the prosthetic replacement, which is a rapidly growing solution for increasingly younger patient populations, with inherent risks and costs both for patients and health care systems [19, 30]. In this light, when patients do not sufficiently respond to conservative treatments, injective approaches may provide the necessary benefit to address the acute phase and allow symptoms to regress to the mean while avoiding more invasive solutions. This study confirmed that the symptomatic trajectory after 36 months showed no worsening in knee OA patients and, once the acute phase was addressed with the injectable options, they remained stable or even improved over time, with only a limited percentage of patients requiring knee replacement.

As an in‐between solution in the vast landscape of options ranging from conservative solutions to TKA, intra‐articular injective therapies represent a well‐accepted approach to address knee OA in the clinical practice [9]. Among the available options, CS and HA represent the most recommended products by major international societies guidelines for knee OA treatment. The American College of Rheumatology (ACR) strongly recommends the use of CS injections, while conditionally recommending against the use of HA and considering it applicable only when other alternatives have been exhausted or failed to provide satisfactory results [28], The European Society for Clinical and Economic Aspects of Osteoporosis and Osteoarthritis (ESCEO) affords a weak recommendation to the use of CS injections in knee OA, stating that CS are more effective than HA in the first few weeks, while affording a weak recommendation to the use of HA in patients having contraindications or not responding to NSAIDs [16]. The Osteoarthritis Research Society International (OARSI) conditionally recommends intra‐articular CS and HA injections, stating that CS may provide short‐term pain relief, whereas HA may have beneficial effects on pain at and beyond 12 weeks [6]. Finally, the American Academy of Orthopaedic Surgeons (AAOS) provides a moderate recommendation on the use of CS and HA for the treatment of symptomatic knee OA, supporting the use of CS for short‐term symptomatic relief and endorsing the use of HA in patients who failed other treatments [15].

Numerous studies focused on the direct comparison of the clinical effectiveness of CS and HA. According to the most updated and comprehensive evidence, the comparison of CS and HA showed no superiority of CS injections for knee OA treatment. In particular, HA obtained a marginally superior pain relief al long‐term follow‐up compared to CS, with a VAS improvement statistically significant but not reaching the MCID [12]. The evaluation of the clinical relevance of a specific treatment is crucial to understanding its real potential in the clinical practice [27]. In fact, a statistically significant improvement does not always reflect a clinically meaningful change for the patient, thus not providing robust evidence of treatment efficacy as a base for guidelines in the clinical practice [20]. Recently, the MCID evaluation has been proposed to understand and interpret the clinical relevance of the benefits provided by treatments, and these values have been quantified also for intra‐articular injections for knee OA. Accordingly, MCID values have been used in the current study as a reference to interpret the clinical relevance of the documented improvement. In this light, CS outperformed HA at short‐term follow‐up, with more patients reaching the MCID. On the other hand, HA was able to produce a clinically relevant improvement in terms of WOMAC at long‐term follow‐up in the intragroup analysis and a VAS improvement that was not far from exceeding the MCID, while the improvements in the CS group were not statistically significant. However, the direct comparison in the intergroup analysis showed only marginal clinical benefits in favour of HA, with the only difference represented by WOMAC improvement, which did not reach the MCID. Overall, these findings appear in line with the recommendations of the major international societies' guidelines, suggesting that CS could provide superior pain relief in the short term, while HA might produce a greater functional improvement in the longer term.

Safety represents another key aspect of the comparison between CS and HA use for knee OA injective treatment. While intra‐articular CS injections are relatively safe and present a low risk of short‐term complications [10, 26], concerns have been raised on their long‐term effects on articular cartilage, as reported by the ACR guidelines [28], with some studies reporting negative effects especially after repeated administration [29, 41]. To this regard, in a saline controlled double‐blind RCT in 140 knee OA patients receiving an intra‐articular CS injection every 3 months for 2 years, the magnetic resonance imaging analysis at the last follow‐up revealed a significantly greater cartilage volume loss in patients treated with CS injections compared to the control group [29]. Similarly, a large observational study on a cohort of 684 patients documented that repeated intra‐articular CS injections were associated with an increased risk of knee OA progression in terms of radiographic KL grade and JSN over a 2‐year follow‐up period [41]. This could suggest an increased risk of knee OA progression in patients receiving repeated CS injections in the clinical setting. At the same time, concerns have been raise also for HA injections, with some studies suggesting a worse safety profile compared to sham or nonintervention control [35]. However, multiple meta‐analyses demonstrated the overall safety of HA [5, 25], even when administered in repeated injections [4].

Despite the possible differences between CS and HA in terms of safety and knee OA progression, which still remain a subject of debate, recent evidence showed that patients receiving only CS or HA presented a similar length of time between the first injection and the TKA associated with the injected joint [24]. The present study confirms the findings reported in the literature showing similar results in terms of radiographic knee OA progression evaluated by KL and JSN between CS and HA, as well as no difference in the number of patients requiring TKA after the injection. Future trials are needed to provide a larger amount of high‐level data allowing to stratify the results according to the specific product used and to the number of injections performed, providing specific indications on the safety and effect on OA progression of CS and HA to tailor the injective therapy to the individual patient's characteristics.

In this sense, it is to be mentioned that the field of intra‐articular injections for knee OA treatment is still characterised by significant heterogeneity and lack of standardisation, not only for the new emerging products but also for the oldest and most used options like CS and HA. In fact, a recent systematic review and meta‐analysis investigating the most updated preclinical and clinical data showed that the evidence on the comparison of different CS types is limited, hindering the possibility of determining the best CS approach in terms of molecule, dose, and formulation for the intra‐articular injection in the clinical practice [11]. Similarly, HA products present a considerable variability in terms of origin, method of production, molecular weight, biologic characteristics, rheologic properties, residence time in the joint, and pharmacodynamic properties [7]. Major international societies guidelines currently do not provide recommendations on this complex but relevant aspect, highlighting the need for further research efforts to identify the product characteristics able to maximise the treatment effectiveness as well as the safety profile of both CS and HA, ultimately optimising the management of knee OA patients in the clinical practice. To this regard, it is also important to underline that both types of products are known to provide a limited benefit and no disease modifying effects [10], while regenerative options are emerging as promising to offer better and longer‐lasting results [12, 13, 14, 32], and future studies should investigate if they could offer a better solution to further improve the OA disease trajectory and postpone invasive solutions more than CS and HA.

This study presents some limitations that require consideration. First, the patients were part of a database, and the outcomes analysed as well as the follow‐ups considered are related to data availability provided by the OAI, which was not designed to compare the two treatments. Second, the design of this study was retrospective, and the lack of randomisation may have influenced the study outcomes due to observer and performance biases, limiting the overall generalisability of the obtained results. Third, the relatively limited number of patients included, especially in the HA group, prevented the performance of subanalyses to further investigate injection‐related aspects. Moreover, most of these patients were affected by early to moderate knee OA, thus are not representative of more severe disease stages. Finally, no data were available to relate the results to specific CS and HA products. Despite these limitations, the present study provided important results, offering valuable information on the use of CS and HA for knee OA treatment, supporting the use of CS to offer short‐term pain relief and of HA to provide longer‐term functional improvement in knee OA patients.

## CONCLUSION

The symptomatic trajectory after 36 months showed no worsening in knee OA patients undergoing intra‐articular injections and different benefits based on the treatments: CS offered clinically relevant benefits compared to HA at short‐term while HA provided superior functional improvement at long‐term, with no differences in terms of radiographic OA evolution, knee swelling, and progression to TKA.

## AUTHOR CONTRIBUTIONS

**Alessandro Bensa**: Conceptualisation; methodology; investigation; supervision; writing—original draft. **Luca Bianco Prevot**: Conceptualisation; data curation; formal analysis; methodology; investigation; visualisation; writing—original draft. **Giuseppe M. Peretti**: Supervision; writing—review and editing. **Giuseppe Filardo**: Conceptualisation; methodology; project administration; supervision; writing—review and editing.

## CONFLICT OF INTEREST STATEMENT

The authors declare no conflicts of interest.

## ETHICS STATEMENT

The OAI study was approved by the institutional review board and all participants signed informed consent forms. The National Institute of Arthritis and Musculoskeletal and Skin Diseases (NIAMS) at the National Institutes of Health (NIH) appointed an independent Observational Study Monitoring Board (OSMB) to oversee the OAI study from 2002 to 2014.

Responsibilities of the board included:

- evaluating the study progress;
- protecting the safety of study participants;
- and considering ethical and other external factors that could impact participant safety.

The OSMB was disbanded upon study completion and monitoring obligations were fulfilled.

## Data Availability

Data used in the preparation of the current article are available for public access at http://www.oai.ucsf.edu/.
